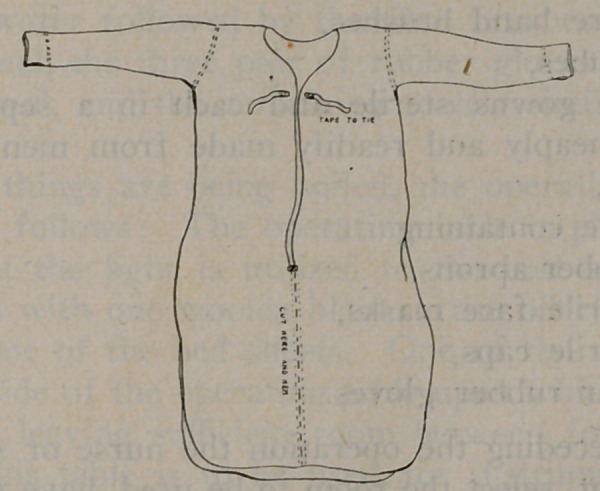# A Simplified Technique in House Operations

**Published:** 1913-05

**Authors:** Julius Richter

**Affiliations:** Buffalo


### A Simplified Technique in House Operations

BY DR. JULIUS RICHTER
Buffalo

THAT this subject is so sparsely treated by all modern authors is not remarkable in view of the many well-governed and easily accessible hospitals, and the growing tendency of our profession to make the public subservient to its wishes.

Every man in active practice knows that the public very often demands that a required operation be performed in the patient's home; that to perform it there is often necessary to the patient's well-being; that we are sadly in need of a simplified course of procedure; and that when a technique has been thought out, very good work can be done under home conditions. The subject is worth an evening's discussion.

There is no surgical procedure of which I know, that cannot be performed in the home, providing the patient can afford the assistants and accessories necessary. An operation in the house should be performed according to hospital technique as nearly as circumstances will permit; but do not allow marble and nickel-plated trimmings to awe you into the belief that ingenuity cannot work without them. The simplicity of the Mayo clinic is in direct proportion to its efficiency. Nor must one forget that a number of assistants in hospital clinics are there for experience and not to fill a necessity. It has often appeared to me that many clinics substitute numbers for competence—million-dollar operating rooms with student anaesthetists.

Then, again, the elaborate instrument displays are ridiculous when one sees so competent an operator as Dr. Robert Morris perform an appendectomy with only scissors and one haemostat. It is not, therefore, a hasty deduction to state that the paraphernalia for this work can be made simple, inexpensive, and portable.

The public have always preferred to be sick at home, if at all, rather than in a strange place. If a disturbed mental state is inimical to a patient's recovery, it seems logical that, other things being equal, he will do better in his home than in a hospital. From an economical standpoint I doubt whether, value for value, he is not a saver by remaining at home even with the added expense of a trained nurse. In cases remote from hospitals, emergency takes precedence over every other consideration. For these cases alone, a surgeon ought to be provided with a technique and outfit for the performance of house operations. The number of trivial minor cases such as finger amputations, circumcisions, adenoids and what not, which are forced into the hospitals by ambitious surgeons, is absurd. One might well feel ashamed to inflict a wage-earner with hospital expense for so small a thing as a felon.

The standpoint of the attending physician must also be candidly discussed. When a large public hospital donated by the people of a free country will rule that no physician or surgeon other than those of the hospital staff shall operate or attend upon his patient, private or otherwise, while in that institution, it

Diagram of Operating Room.

would seem that self preservation requires outside practitioners to keep their patients out of hospitals. Competent operators DO exist outside hospital staffs, and their only alternative is in the house operation.

It follows from the above that the house operation is a needed work and we will proceed to a consideration of its technique.

There are a few absolute essentials without which one cannot well dispense. (1)—A warm, well-lighted room to serve as the operating room. This room should be situated near the running water and the source of heat for sterilization. . The kitchen is a preferable place, but often an upper room near the bath gives better light. Artificial light, I might say here, is often sufficient, especially if the lights have reflectors. The room should be such as can be thoroughly cleaned, but do not let its unhygienic state deter you in the confident effort at aseptic work. I doubt whether any room has more virulent pus spilled about it at times than the hospital clinic, yet you work there with impunity. On the other hand, obstetric operations, are daily performed by you, especially among the foreign population, in the most filthy environment with remarkably little infection. The gist of it is that healing by first intention depends more on the asepsis of the operator and field of operation than on the environment. (2)—A trained nurse to assist at the operation and attend the patient thereafter. In the minor cases her after attendance may be dispensed with. She ought to be of a practical turn of mind, familiar with hospital technique, and preferably one with

Suit Case Packed for Opĕration.

whose work you are acquainted. (3)—A surgical asssistant and an anaesthetist, in both of whom you place complete cofidence. (4)—The necessary paraphernalia, which includes:

Surgical instruments.
Utensils.
Accessories.

It would be futile to name the surgical instruments required, as each man has his method and the particular procedure you institute will be described and the instruments enumerated in any first-class operative surgery. Although the outlay for instruments is a necessary one, it is often remarkable how ingenuity will overcome the need of complex mechanisms.

The utensils and accessories comprise those to be supplied by the patient and those to be supplied by the surgeon.

The patient will supply the following utensils:

2 wash boilers,

1 long-handled dipper or ladle for hot water,

1 kitchen table measuring two by four feet to be used as an operating table,

2 kitchen tables of about the same size,
1 small table or stand about 2x2 feet,
2 wooden kitchen chairs,
2 wooden or china pails,
3 china or enameled pitchers, each holding at least three quarts,
1 bed pan and
2 wooden boxes or stools about 18 inches high.

The patient will also furnish the following accessories:
3 clean sheets,
36 soft towels, closely woven,
2 woolen blankets,
1 piece of oil cloth or rubber sheeting, and will get the following from the apothecary's:
2 half pound cans of ether and four ounces of chloroform,

Serviceable operating gowns can be readily made from men's cotton night shirts. The shirt is cut, hemmed, tapes attached wherewith to tie, and the garment worn as any operating gown.

1 pint alcohol,
1 roll zinc adhesive two inches wide, and
1 cake ivory soap.

The operator will supply the following utensils:
4 large enameled basins with flat bottoms,
4 shallow enameled butcher's trays measuring about 15x30 inches,
1 deep enameled tray measuring about 6x12 inches,
3 small enameled custard cups of different sizes to nest into each other, and
1 pair canvas or leather leg holders.

The operator will furnish the following accessories:
6 dozen sterile gauze sponges in packages of one dozen,
8 sterile large gauze packs measuring a yard square,
1 sterile package containing complete dressing as follows:
  1 dozen gauze compresses cut 18x18 inches, and folded to measure 6x6 inches.

1 large pad of absorbent cotton (a piece 18 inches long cut from a roll and folded once over),
1 many-tailed bandage of cotton cloth,
4 roller bandages four inches wide,
1 dozen large safety pins.

1 four ounce bottle tincture of iodine,
1 six ounce bottle hydrogen peroxide,
1 two ounce bottle 10 per cent. camphor in olive oil,
1 hypodermic syringe with accompanying tablets of
Morphine sulphate, 1/8 gr.,
Nitroglycerine, 1/100 gr.,
Strychnine sulphate, 1/60 gr.

The required suture material. (Catgut in sealed glass tubes is preferable. These are kept in a fruit jar with sufficient carbolic acid to cover them).

1 dozen fibre hand brushes,
Drainage tubes,
4 operating gowns sterile and each in a separate package. (These are cheaply and readily made from men's large cotton night gowns),
One package containing
2 rubber aprons,
3 sterile face masks,
3 sterile caps,
3 pair rubber gloves.

The day preceding the operation the nurse or surgeon should visit the patient, select the room to be used, have removed therefrom all curtains, pictures and unnecessary furniture, have the woodwork wiped with damp clothes and the floor scrubbed. In emergency cases, where one is to operate at once, it is unwise to allow the cleaning process to contaminate the air with dust. The two kitchen chairs are scrubbed, the tables and pails cleaned and all placed in the room.

One of the wash boilers is thoroughly cleaned, filled with clean water, the dipper placed within, the whole covered and boiled for ten minutes. While this last is in progress the patient may be prepared.

The patient should have taken, or should have been given a full bath and have had the site of operation shaved and scrubbed. A towel may be boiled for a few minutes, wrung out of a weak bichloride, placed upon the area and held in position by another towel or a bandage. A cathartic may or may not be given according to the proposed operation. In work upon the perineum, rectum, etc., it is the better practice to give the cathartic a day before and give an enema on the morning of operation. The reason is obvious. With instructions to the patient to forgo food, he may be left until the morrow.

Before leaving the patient's house, the wash boiler with boiling water should be set aside to cool and the other wash boiler should be cleaned, filled with clean water, covered, and instructions given to have it actively boiling at a given time the following morning.

The nurse or surgeon upon arriving next morning will proceed as follows: The linen and silk-worn gut sutures are threaded on the needles required and placed securely on a folded towel by several bites of the needle. The towel is then folded to cover the sutures, and it, with the drainage tubes and instruments, is wrapped in another towel and the whole then lowered into the boiling water of the wash boiler. Next the basins, pitchers, butcher's trays, deep tray, and custard cups are placed in the boiling water followed by the fibre brushes wrapped and pinned in a towel, the three pair of rubber gloves wrapped and pinned in a towel, and the remaining towels similarly wrapped and pinned.

While these things are being boiled, the operating room may be arranged as follows: The operating table is placed near the window so that the light is utilized to the best advantage. It is then covered with one woolen blanket, the oil cloth or rubber sheeting, and one of the bed sheets. One of the other tables is placed at that side of the operating table upon which the surgeon elects to work, leaving sufficient room between for convenience of motion. This table is to be used for instruments, sponges, two basins, sutures, etc. A pail is placed upon the floor between these tables for waste material. The remaining table is now placed at one side of the room and covered by the other bed sheet. This table is to be used for supplies. Beside it are placed the other pail and the wash boiler full of sterile water. The small stand is placed opposite the last table and is used as a wash stand.

Having thus arranged the operating room, the wash boiler containing the utensils is now brought in and, by means of placental forceps, the various articles are withdrawn and placed as follows: The butcher's trays are placed side by side upon the instrument table thus affording a sterile surface for instruments, sponges, etc. Two basins, one filled with warm sterile water and the other with warm, weak bichloride solution, are next placed there. Into the bichloride are placed the towels, well drained, and the gloves. One towel is wrung out, placed on the supply table, and on it is placed the brushes. The instruments are now brought out, drained, and placed on a tray; the custard cups on a tray; the pitchers on the supply table; and the remaining two basins on the wash stand, one filled with warm sterile water and the other with warm, weak bichloride solution.

The wash boiler is emptied, refilled with clean water, brought to a boil, and placed next to the one with cool sterile water. Six teaspoonfuls of salt are placed in a sauce pan with a cup of water and boiled for a few minutes. Half of this solution is poured into one pitcher, and half into another. One is filled with three quarts hot and the other with three quarts cold sterile water. By mixing the contents in the third pitcher, normal salt solution of any temperature may be produced.

The nurse now opens the packages containing gowns, masks, caps, etc., upon the supply table, being careful lest she contaminate the contents. Having done this, she scrubs up, receives a cap, mask and gown, and puts on her rubber gloves. She now spreads the instruments, drainage tubes and needle towel conveniently. She then, with thumb forceps, withdraws from the fruit jar, which the surgeon opens and holds, the required number of catgut tubes. These she places in the deep tray and the surgeon pours in enough alcohol to cover them. The catgut tubes are now in condition to be handled. The surgeon next fills one custard cup with tincture of iodine, and one with peroxide of hydrogen.

The patient is now brought in, dressed in night gown and stockings, and is placed as follows:

### For Abdominal Operations.

The patient is placed upon the table so that the knees bend over the table's end. It is well to have the woolen blanket folded thick over the table's end to avoid undue pressure on the under aspect of the knee. The patient's ankles are strapped firmly to respective legs of the table and his feet are allowed to rest on the boxes. The anaesthetic is then begun and the patient's hands pinioned under him.

The assistant scrubs up and, assisted by the surgeon, dons cap, mask, gown, and gloves. The surgeon removes the dressings from the patient's abdomen and the assistant, by means of a gauze sponge held in a haemostat, paints the abdomen liberally with tincture of iodine. Then with the warm bichloride towels thoroughly wrung out, he covers the patient. This procedure of covering the patient with wet towels will be objected to by some. It will be remembered, however, that but a few years ago it was the general routine. If the room temperature is kept sufficiently high, there need be no fear of excessive body-heat radiation.

The patient being thus prepared, the surgeon scrubs up and dons his cap, mask, gown and gloves. He purposely is last to do this in order that he, himself, may correct any irregularity in arrangement or procedure. He now takes his place and the operation proceeds. The assistance of a second nurse is obviated by having the arrangements as described. Should it become necessary to handle an unsterile object, a sterile towel is placed over the hand and the towel discarded after use.

If the Trendelberg position of the patient is needed, it is produced as follows: The anaesthetist braces his feet against the table-legs, drawing the upper part of the table-legs toward him. At the same time the surgeon and assistant grasp the opposite end of the table, protecting their gloves with sterile towels, and raise that end while the nurse shoves the boxes under the table-legs.

### For Other Operations.

In operations upon the perineum or rectum, the legs are held by means of the leg holders. Should a combined operation, vaginal and abdominal, be performed, the surgeon and assistant change the patient's position and then change their gowns and gloves, the extra ones having been provided. In operations upon other parts of the body, ingenuity must suggest the minor procedures.

The after care differs in no wise from that in the hospital. The greatest difficulty in the after management is from the well meant but generally misdirected efforts of friends and family. The nurse should be in supreme command and brook no interference from anyone. You will find that all in all, your patients recover just as often and with as happy a state of mind as in hospital practice

---

Sadistic Murder and Suicide. November 3, 1912, the body of a prostitute was found in the Bois de Boulogne. Post mortem examination showed the ordinary signs of strangulation. A peculiar and, at first, unexplained fact, was that a piece of skin, apparently human, was found between the victim's teeth. The same day a count was found dead, and with the body was a note dated the day previously expressing his intention of committing suicide immediately. But, according to the classic signs, death must have occurred November 3. Examination revealed a lacerated wound of the prepuce, with a part missing. The publicity of these two post mortem examinations led to a comparison of notes, and it was found that the piece of skin mentioned fitted the wound. Apparently, the count wished to prove an alibi by antedating his note regarding the time of suicide.

## Figures and Tables

**Figure f1:**
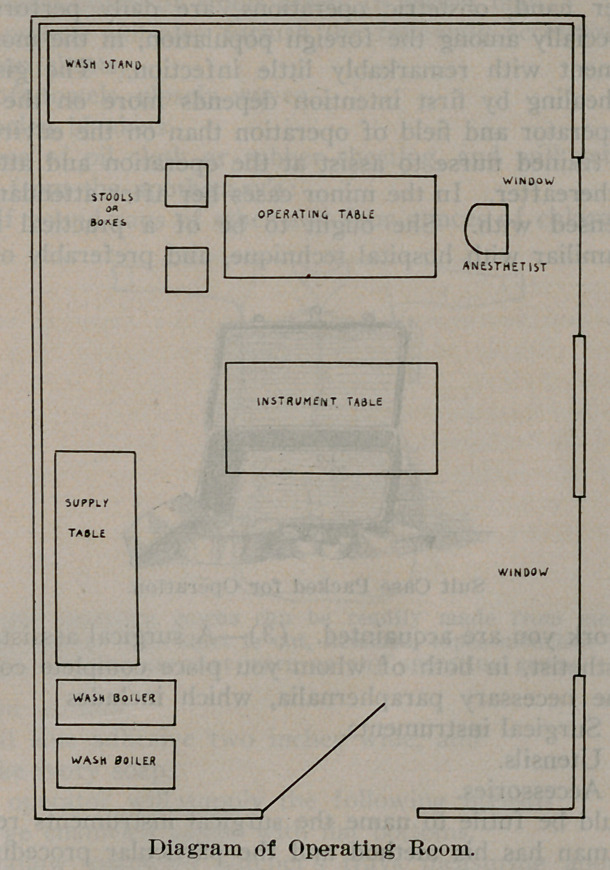


**Figure f2:**
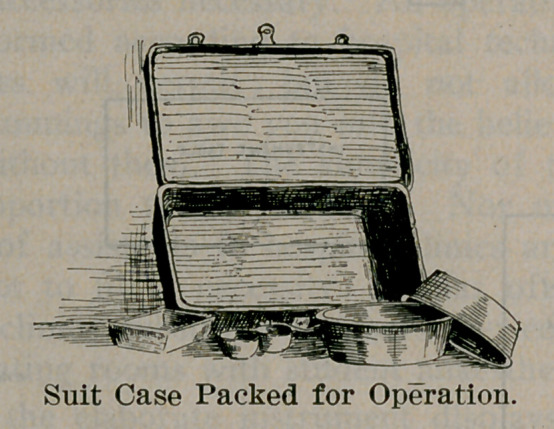


**Figure f3:**